# Supplementary material for: Global impact and cost-effectiveness of one-dose versus two-dose human papillomavirus vaccination schedules: a comparative modelling analysis
Source: BMC Med. 2023 Aug 28;21:313. doi: 10.1186/s12916-023-02988-3 (PMC10463590; doi:10.1186/s12916-023-02988-3)
Supplement: Supplementary file 1 — Additional file 1: Table 1. Transmission dynamic model description. Table 2. Vaccination strategies. Table 3. World Bank income group. Table 4. Inputs: HPV-FRAME reporting standard checklist. Table 5. Inputs: HPV-FRAME reporting standard checklist (Continued). Table 6. Outputs: HPV-FRAME reporting standard checklist. Table 7. Internal validation. Figure 1. Cumulative cervical cancers averted by routine one-dose HPV vaccination by income groups, no discounting. Figure 2. Cervical cancer deaths averted by routine one-dose HPV vaccination by income groups, no discounting. Figure 3. Cervical cancers averted by routine one-dose HPV vaccination by income groups, discounted. Figure 4. Cervical cancer deaths averted by routine one-dose HPV vaccination by income groups, discounted. Figure 5. Cumulative cervical cancers averted by routine one-dose HPV vaccination by income groups, discounted. Figure 6. Proportion of cervical cancers averted by 1-dose compared to a perfect vaccine, discounted. Figure 7. Proportion of cervical cancer deaths averted by 1-dose compared to a perfect vaccine. Figure 8. Cervical cancers averted by routine one-dose HPV vaccination by income groups with a 2-valent vaccine. Figure 9. Cervical cancers averted by routine one-dose HPV vaccination by income groups at lower coverage. Figure 10. Proportion of cervical cancer deaths averted by 1-dose compared to a perfect vaccine at lower coverage. Figure 11. Proportion of cervical cancer averted by 1-dose compared to a perfect vaccine when vaccination is delayed. Figure 12. Cervical cancers averted by routine one-dose HPV vaccination by income groups at when vaccination is delayed. Figure 13. Threshold cost to pay for the first and second dose of vaccine, discounting on health outcomes and costs. Figure 14. Threshold cost to pay for the first and second dose of vaccine, no discounting. Figure 15. Number of girls needed to be vaccinated to avert one additional case over the years 2021–2120. [file 12916_2023_2988_MOESM1_ESM.pdf]

Global impact and cost-effectiveness of one-dose versus two-dose human papillomavirus vaccination schedules: a comparative modelling analysis

Supplementary Material

Kiesha Prem[*], Yoon Hong Choi[†], Élodie Bénard[†], Emily A Burger[†], Liza Hadley, Jean-François Laprise, Mary Caroline Regan, Mélanie Drolet, Stephen Sy, Kaja Abbas, Allison Portnoy, Jane J Kim, Marc Brisson, Mark Jit[‡]

Contents

1 Materials and methods **1**
1.1 Summary of evidence on protection from one-dose HPV vaccination . . . 1
1.2 Model description . . . 2
1.3 The HPV-ADVISE models . . . 2
1.4 The Harvard model . . . 3
1.5 The UK Health Security Agency (HSA) model . . . 3
1.6 Papillomavirus Rapid Interface for Modelling and Economics (PRIME) model . . . 4
1.7 Vaccination strategies . . . 4
1.8 World Bank income groups . . . 4
1.9 Population projection . . . 4
1.10 HPV-FRAME reporting standard checklist . . . 6

2 Results **10**
2.1 Cervical cancers cases and deaths averted . . . 10
2.2 Cervical cancer cases and deaths: discounting health outcomes at 3% . . . 10
2.3 Threshold costs . . . 24
2.4 Number needed to vaccinate . . . 27
2.5 Internal validation . . . 29

1 Materials and methods

1.1 Summary of evidence on protection from one-dose HPV vaccination

The KEN SHE trial (Barnabas et al., 2022) found that one-dose HPV vaccination was >95% effective in preventing the onset of persistent HPV 16/18 infections. This finding was supported by nonrandomised observational data in independent trials: the Costa Rica HPV Vaccine Trial (CVT) (Kreimer et al., 2020) and the IARC India HPV trial (Basu et al., 2021); the posthoc analyses showed that one-dose vaccination is as effective in preventing HPV infections as multidose vaccination in healthy young females up to 11 years post-vaccination. The CVT and IARC India HPV trials found that efficacy against HPV 16 and 18 infections

[*]Correspondence to kiesha.prem@lshtm.ac.uk.
[†]Contributed equally
[‡]Correspondence to mark.jit@lshtm.ac.uk.

was comparable in one-dose and multidose schedules. The DoRIS study (Watson-Jones et al., 2022) found that while antibody titres were lower with one dose than with two or three doses, they were significantly higher in one-dose HPV vaccine recipients compared to natural infection. Antibody titres in one-dose arms remained stable throughout follow-up, up to 11 years post-vaccination.

1.2 Model description

The three transmission dynamic models listed in Table 1—HSA, HPV-ADVISE, Harvard—were developed independently, but have several common features (Brisson et al., 2012, 2017, 2020; Burger et al., 2018; Campos et al., 2014; Choi et al., 2010; Van De Velde et al., 2012). The models stratify population by age, sex and sexual activity-based risk group, and screening behaviour-based risk group. They capture HPV natural history and disease, as well as HPV transmission as informed by country-specific sexual behaviour surveys.

Table 1: Transmission dynamic model description.

Model configuration	HSA	HPV-ADVISE	Harvard
Countries considered	United Kingdom	India, Nigeria, Uganda, Vietnam	United States, Uganda, Nicaragua, El Salvador
Age cohorts	Cohorts of 10–74 years old females and males	Open, stable population from 10-years-old until death	Cohorts of 10–99 years old females and males
Routine vaccination	10-year-old girls	10-year-old girls	10-year-old girls
First-year catch-up campaign	11–14-year-old girls	11–14-year-old girls	11–14-year-old girls
Simulations	Probabilistic sensitivity analysis using second-order simulation methods		
Cancer outcomes	Age-specific cervical cancer incidence		

We use a hybrid approach: first, we consider the age-specific impact that HPV vaccines may have using the results of the three independent HPV transmission dynamic models across 10 settings (Brisson et al., 2012, 2017; Burger et al., 2018; Campos et al., 2014; Choi et al., 2010; Van De Velde et al., 2012), and second, extrapolate these effects to the remaining countries in the world using data on population demographics and cervical cancer burden synthesised in a static model Papillomavirus Rapid Interface for Modelling and Economics (PRIME) model (Abbas et al., 2020; Jit et al., 2014).

1.3 The HPV-ADVISE models

The HPV-ADVISE (Brisson et al., 2012, 2017) and HPV-ADVISE LMIC (Drolet et al., 2021) models are individual-based, transmission-dynamic models of multi-type HPV infection–with 18 HPV types modelled separately, including vaccine-preventable types 6/11/16/18/31/33/45/52/58—and diseases. Designed to examine HPV vaccination policy questions in low- and middle-income countries (LMICs) settings, HPV-ADVISE LMIC has the same underlying structure for all LMICs and the same basic model structure as HPV-ADVISE for high-income countries (HICs) but modified to capture differences in sexual behaviour between LMICs and HICs. More details can be found in the Technical Appendices: http://www.marc-brisson.net/HPVadvise.pdf and http://www.marc-brisson.net/HPVadvise-LMIC.pdf. HPV-ADVISE and HPV-ADVISE LMIC reproduce demographic characteristics, sexual behaviour and transmission of HPV, the natural history of HPV-associated diseases (HPV infection, natural immunity, three grades of cervical lesions, and three cervical cancer stages), screening and treatment. Transmission is gender- and age-specific, and depends on sexual behaviour (e.g., mixing patterns) and HPV biology and natural history (e.g., probability of transmission and natural immunity). For each country, we identified 50 parameter sets

that simultaneously fit highly stratified country-specific sexual behaviour and HPV epidemiological data obtained from published articles, specific studies, and international population-based datasets (HPV-ADVISE LMIC Technical Appendix Table A1). These 50 parameter sets represent uncertainty in model parameters and variability in sexual behaviour and HPV epidemiology within the modelled country. They were used to generate age-specific incidence of cervical cancer over time for each vaccination scenario investigated. HPV-ADVISE projection of age-specific incidence is generated in 5-year age groups from 10 to 64 years of age with an additional age group for 65 years of age and over. This projected incidence is mapped to 1-year age strata for use with the PRIME model.

1.4 The Harvard model

The Harvard model (Burger et al., 2018; Campos et al., 2014) uses a multi-model approach to project the population health consequences of alternative cervical cancer scenarios over time. The multi-modelling approach involves two components: (1) Harvard-HPV, a dynamic, compartmental model of natural history that simulates the potential health outcomes of nine HPV infection genotypes and HPV sexual transmission between males and females; and (2) Harvard-CC, a static, individual-based model of HPV-induced cervical cancer. We simulated HPV transmission in Harvard-HPV as a function of partnership acquisition and dissolution, by sex, age, and sexual activity level. HPV infection can be transmitted, depending on the number of new partners, partner infection status, probabilities of HPV transmission given contact with an infected partner, and duration of the partnership. Individuals develop type-specific natural immunity when they clear an HPV infection, which reduces their susceptibility to future same-type infection. Harvard-HPV was calibrated to reflect variations in genotype- and sex-specific transmission probability, and genotype and sex-specific natural immunity for two sexual behaviour settings (low- and high-HPV prevalence). For these two settings, reductions in HPV incidence by genotype over time associated with each control strategy compared with no intervention; these reductions served as inputs into Harvard-CC. Using Harvard-CC, we project cervical cancer incidence by age over time for each scenario as a series of transitions through health states that describe true underlying health, including HPV infection, precancerous states, and invasive cancer. Women who develop cervical cancer may be detected symptomatically or progress to a more severe cancer stage. The model is adapted to different epidemiological settings—the United States, Uganda, El Salvador and Nicaragua—by fitting or calibrating the model using the best available country-specific data, e.g., HPV prevalence, HPV type distribution in CIN 2, CIN3 and cervical cancer. To capture parameter uncertainty, the reductions in HPV incidence associated with 50 best-fitting dynamic transmission model parameter sets (for the two epidemiological settings) were propagated through four cervical carcinogenesis models that have been previously calibrated (i.e., fit) to the United States, Uganda, El Salvador, or Nicaragua. Consequently, the current analysis projections reflect the uncertainty of the vaccine on HPV incidence reductions but not uncertainty in the natural history of cervical carcinogenesis, which may underestimate our uncertainty bounds. All models, except the US model, were simulated in the absence of screening. For the US model, we assumed cytology-based screening for women aged 21-65 years under current screening practice patterns, as we have assumed previously (Kim et al., 2021).

1.5 The UK Health Security Agency (HSA) model

The UK Health Security Agency model (Choi et al., 2010) is a parameterised family of dynamic transmission models of heterosexual HPV transmission and HPV-related diseases—cervical dysplasia, cervical cancer and anogenital warts—in the United Kingdom (UK). At equilibrium, the model population consists of 49 million people. They are divided into birth cohorts between 10 and 74 years old, with an equal split between males and females. Age- and sex-specific mortality rates were obtained from the Office for National Statistics of the UK. The model population is closed, and it assumes no immigration or emigration. This series of transmission models represent different parameters about HPV biology and epidemiology, including sexual behaviour, natural immunity, vaccine characteristics, disease progression and screening accuracy with thousands of combinations of assumptions on the parameters generated and then fitted to prevalence data. Each scenario or combination of assumptions was then fitted to epidemiological data and the best-fitting scenarios used to predict the impact of vaccination.

1.6 Papillomavirus Rapid Interface for Modelling and Economics (PRIME) model

The Papillomavirus Rapid Interface for Modelling and Economics (PRIME) model (Abbas et al., 2020; Jit et al., 2014) is a WHO-supported model to estimate the health impact—cervical cancer cases, deaths, or disability-adjusted life-years averted—and cost-effectiveness of HPV vaccination strategies among adolescent girls. As a static model, PRIME does not consider herd effects and cross-protection against non-vaccine HPV types and thus provides conservative estimates of the vaccine impact. However, it adjusts for lower vaccine protection in vaccinated individuals who have sexually debuted before vaccination. The updated PRIME (Abbas et al., 2020) uses population demography of the United Nations World Population Prospects 2019 revision (United Nations Department of Economic and Social Affairs Population Division, 2019) to incorporate population ageing. In the updated model, cervical cancer burden was updated from the International Agency for Research on Cancer estimates for Global Cancer Incidence, Mortality and Prevalence (GLOBOCAN) 2012[1] database to GLOBOCAN 2018 database (Bray et al., 2018), and disability weights were updated from estimates of the Global Burden of Disease 2001 study[2] to estimates of the 2017 study[3]. PRIME can also estimate the health impact of bivalent or quadrivalent and nonavalent vaccination programmes. Disability-adjusted life year (DALY) was estimated as the sum of years of life lost due to premature mortality and years of life lost due to time lived in states of less than full health due to disability (more details in Appendix Tables A2.1. and Tables A2.2. in Abbas and colleagues Abbas et al. (2020)). The disability weights for different phases (diagnosis and primary therapy, controlled, metastatic, terminal) of cervical cancer from the Global Burden of Disease 2017 study were used to estimate the years of life lost due to disability. The country-specific life tables were used to estimate the years of life lost due to premature mortality. The treatment cost, including facility, staff, medical device and pharmaceutical costs, for cancers detected at each stage in 14 WHO-CHOICE regions was obtained from a WHO-CHOICE study[4].

1.7 Vaccination strategies

We model routine annual vaccination with the 9-valent (or 2-valent) vaccine in 10-year-old girls to begin in 2021 and run uninterrupted until 2120. We also include a catch-up of girls up to age 14-year-old in the first year of the programme. Vaccine coverage was assumed to be 80%. We measure and compare population-level impact (e.g., cervical cancers averted, number of females needed to be vaccinated, threshold costs of the first and second dose of the vaccine) for three vaccine strategies: no HPV vaccination; a one-dose HPV vaccination schedule in which we assume that one dose of the vaccine gives either a shorter duration of protection (20 or 30 years) or lower vaccine efficacy (e.g., 80%) compared to two doses; and a two-dose HPV vaccination schedule in which two doses of the vaccine would provide lifetime protection. Although the Harvard, HPV-ADVISE and HSA models incorporated vaccine efficacy differently—vaccine 'degree' for the Harvard model (calibrated to 86%) and vaccine 'take' for HPV-ADVISE and HSA—the models achieved an 80% cumulative reduction in vaccine-type HPV infections for a vaccinated cohort at year five.

1.8 World Bank income groups

The World Bank assigns countries in the world to three income groups—low, middle, and high-income countries—based on gross national income per capita in current USD of the previous year. Table 3 lists the 188 countries (as ISO 3 country codes) included in the study by their income group.

1.9 Population projection

In this analysis, we model health outcomes in females born in the years 2011–2110. We accrue all health benefits of HPV vaccination up to the end of the routine vaccination programme (i.e. the year 2120) or age 100 of all vaccinated cohorts (i.e., up to the year 2210). The United Nations Population Division projects the population for all countries and areas of the world up to the year 2100 (United Nations Department of

[1]Ferlay et al. GLOBOCAN 2012 http://globocan.iarc.fr/
[2]Mathers CD, Lopez AD, Murray CJ. (2001)
[3]James et al. (2018) Lancet;392(10159):1789–858
[4]Ginsberg et al. (2009) Vaccine;27(43):6060–79

Table 2: Vaccination strategies.

Parameters	Description
Vaccine type	HPV types prevented by vaccination: all high-risk HPV types in the 9-valent vaccine (16, 18, 31, 33, 45, 52, and 58)
Age of vaccination	Age of routine vaccination and ages covered by multi-age cohort (MAC) in first year: Routine 10-year-old girls including first year MAC up to age 14 years old girls
Coverage of vaccination	Proportion of girls in targeted age groups who are vaccinated: 80%
Years of vaccination	Year 1–101 (or calendar years 2021–2120); year 0 is pre-vaccination
Gender	Females
Vaccination scenarios	**Scenario 0:** no vaccination<br>**Scenario 1:** lifetime protection, vaccine efficacy (VE): 100%, i.e., similar to the current two-dose assumptions<br>**Scenario 2:** one-dose offers 20 years protection, VE: 100%<br>**Scenario 3:** one-dose offers 30 years protection, VE: 100%<br>**Scenario 4:** one-dose offers lifetime protection, VE: 80% protection against persistent infection at 5-year time point.

Economic and Social Affairs Population Division, 2019). We use these projections up to 2100, and we then project the population for all countries for the years 2101–2210. We ran a demographic model to age the population in 2100, depleting it by deaths and replenishing it with births.

Fertility rate model: For the demographic model, we used projected five-year age-specific fertility rates for the 188 countries over the period 2095 to 2100, obtained from the United Nations Department of Economic and Social Affairs (United Nations Department of Economic and Social Affairs Population Division, 2019), as a baseline. The age-specific fertility rates were then held fixed in the model to the year 2210.

Because much about the evolution of fertility rates after reaching replacement levels remains unknown and may vary due to cultural differences between countries, we did not seek to extrapolate the fertility trend beyond the latest available projections. Instead, we kept the age-specific fertility rate at 2095 constant until 2210, possibly overestimating fertility as a result. Using the UN's projected sex ratio at birth over the period 2095 to 2100, we distributed the expected births to males and females. We denote b_m to be the proportion of births that are male in country c.

Letting ϕ_a^c be the fertility rate for women aged a in country c, and $F_a^c(t)$ be the number of women aged a in country c in year t, we derived the expected number of births in each country and year to be

$$E^c(t) = \sum_a \phi_a^c F_a^c(t).$$

The number of children surviving to age one was derived by separately calculating the expected number of male births

$$B^c(t) = E^c(t) b_m$$

and female births

$$G^c(t) = E^c(t)(1 - b_m).$$

Mortality rate model: Projected annual, sex-specific, five-year mortality rates were available for the years 2095 to 2100 from the life tables obtained from the United Nations Department of Economic and Social Affairs. The life tables by sex were up to age 100, and they provide projections of the mortality experience of

Table 3: World Bank income group.

Income group	Country code
Low-income	AFG, BDI, BFA, CAF, COD, ERI, ETH, GIN, GMB, GNB, HTI, LBR, MDG, MLI, MOZ, MWI, NER, PRK, RWA, SDN, SLE, SOM, SSD, SYR, TCD, TGO, TJK, UGA, YEM
Middle-income	AGO, ALB, ARG, ARM, AZE, BEN, BGD, BGR, BIH, BLR, BLZ, BOL, BRA, BTN, BWA, CHN, CIV, CMR, COG, COL, COM, CPV, CRI, CUB, DJI, DOM, DZA, ECU, EGY, FJI, FSM, GAB, GEO, GHA, GNQ, GRD, GTM, GUY, HND, IDN, IND, IRN, IRQ, JAM, JOR, KAZ, KEN, KGZ, KHM, KIR, LAO, LBN, LBY, LCA, LKA, LSO, MAR, MDA, MDV, MEX, MKD, MMR, MNE, MNG, MRT, MYS, NAM, NGA, NIC, NPL, PAK, PER, PHL, PNG, PRY, PSE, RUS, SEN, SLB, SLV, SRB, STP, SUR, SWZ, THA, TKM, TLS, TON, TUN, TUR, TZA, UKR, UZB, VCT, VEN, VNM, VUT, WSM, ZAF, ZMB, ZWE
High-income	ARE, ATG, AUS, AUT, BEL, BHR, BHS, BRB, BRN, CAN, CHE, CHL, CYP, CZE, DEU, DNK, ESP, EST, FIN, FRA, GBR, GRC, GUM, HRV, HUN, IRL, ISL, ISR, ITA, JPN, KOR, KWT, LTU, LUX, LVA, MLT, MUS, NCL, NLD, NOR, NZL, OMN, PAN, POL, PRI, PRT, PYF, QAT, ROU, SAU, SGP, SVK, SVN, SWE, SYC, TTO, URY, USA

a hypothetical group of infants born at the same time and subject throughout their lifetime to the specific mortality rates of the years 2095–2100. These were also available for the 188 countries. The mortality rate was assumed constant over five-year intervals of age (with those below 1-year-old having their own mortality group, and those above 100-years-old aggregated in one age group). Using the country-specific life tables, we aged the population forward in time and depleting it by deaths estimated using the age- and country-specific mortality rates.

1.10 HPV-FRAME reporting standard checklist

The checklists presented in Tables 4–6 include the reporting standards from HPV-FRAME (Canfell et al., 2019).

Table 4: Inputs: HPV-FRAME reporting standard checklist.

Domain	Input	Reported by age? (Y/N)	Report by sex (F-only, M-only or both)?	Comments
**Core reporting standards**				
CRS	Target population for intervention	Y	F-only	HPV vaccination of girls aged 10 years; single year of catch-up aged up to age 14 years.
CRS	Sexual behaviour	Y	Y	Reported by age, sex and risk group[1–7]
CRS	Cohort examined for evaluation/time horizon	Y	F-only	101 year time horizon (2020–2120)
CRS	Quality of life assumptions	N/A	N/A	Reported outcomes were cancer cases, deaths, the number needed to vaccinate and threshold costs.
CRS	Calibration	Y	Y	HPV-ADVISE and Harvard models reproduce Globocan 2018 incidence at a country level. The models were calibrated to sexual behaviour, HPV prevalence and cervical cancer incidence[1–7]
CRS	Validation	Y	F-only	Reported in [1–7]
CRS	Costs	N	F-only	Costs of treatment and vaccine reported in [8,9]

Table 5: Inputs: HPV-FRAME reporting standard checklist. (Continued)

Domain	Input	Reported by age? (Y/N)	Report by sex (F-only, M-only or both)?	Comments
**Reporting standard for HPV vaccination in adolescent females**				
1	Vaccine uptake	Y	F-only	Described in Methods of the paper
1	Vaccine efficacy	Y	F-only	Assumed invariant by sex and age
1	Vaccine duration and waning	Y	F-only	Assumed invariant by sex and age
1	Vaccine and delivery costs	Y	Y	Vaccine and delivery costs reported in [8,9]
1	Pre-vaccination disease burden (including PAFs)	Y	F-only	Reported in [1−7]
1	Heterogeneity in sexual behaviour	Y	F-only	Reported in [1−7]
1	Duration of natural immunity	Y	F-only	Reported in [1−7]
**Reporting standard for HPV vaccination using alternative dose schedules**				
7	Vaccine efficacy/waning (by dose, type)	Y	F-only	Assumed invariant by sex and age. We modelled full protection and 80% vaccine efficacy against HPV 16/18/31/33/45/52/58 (for 1-, 2-dose)
7	Timing between doses (for 2-dose)	N/A	N/A	Six months for the 2-dose regimens only given to girls aged 10 years in modelled scenarios.
7	Vaccine cross-protection (by dose, type)	Y implicitly	F-only	We modelled vaccination with a 9-valent vaccine where we assumed that vaccine efficacy is either 80% or 100% for HPV 16/18/31/33/45/52/58.
7	Cost per dose/per vaccinated individual	N	N/A	Cost per dose assumed constant by age within each country.

Table 6: Outputs: HPV-FRAME reporting standard checklist.

Domain	Output	Reported by age? (Y/N)	Report by sex (F-only, M-only or both)?	Comments
**Core reporting standards**				
CRS	Cancer incidence, mortality, life years, QALYs/DALYs (as appropriate)	N	F-only	Reported outcomes were cancer cases and deaths reported combined across all ages as a total over the lifetime of females born 2011–2110 (presented in Fig 2 and the Results of the paper).
CRS	HPV prevalence, pre-intervention CIN2 detected	N	N	We did not report this level of detail as our study focuses on the impact of cancer occurrence and deaths. Impact of interventions on HPV prevalence and CIN2 was thus not a focus of the paper.
CRS	Sensitivity analysis on key inputs	Y implicitly	F-only	In this study, we compared three models with different structural and parameterisation assumptions; hence sensitivity analysis is built into the reported ranges of results between models. Additional details of the sensitivity analysis can be found in the Methods of the paper.
CRS	Incremental cost-effectiveness ratios and costs saved	N	F-only	Threshold costs per dose were reported.
**Reporting standard for HPV vaccination in adolescent females**				
1	Absolute reductions in HPV infections, cervical and other HPV- related cancers and/or warts, post-vaccination	N	F-only	Reported outcomes were cervical cancer cases and deaths, not reductions in HPV infections, other HPV-related cancers or warts. Theoutcomes were combined across all ages.
1	Absolute reductions in CIN2+ post-vaccination	N	N	This paper only focuses on the reduction of cervical cancer cases, deaths and DALYs post-vaccination.
1	Absolute reductions in invasive cancer post-vaccination	N	N	Reported outcomes were cancer cases and deaths combined across all ages or as a total over the lifetime of females born 2011–2110 (year by year : Fig 2) and for females over the vaccination period 2021–2120 (year by year: Fig 2).
**Reporting standard for HPV vaccination using alternative dose schedules**				
7	Threshold cost per dose	N	F-only	Threshold costs per dose were combined across all ages or as a total over the lifetime of females born 2011–2110.

2 Results

2.1 Cervical cancers cases and deaths averted

Figure 1 presents the cumulative cervical cancers cases that could have been averted by routine one-dose HPV vaccination in 188 countries over the years 2021–2120. Figure 2 shows the cervical cancers deaths that could have been prevented by routine one-dose HPV vaccination in 188 countries over the years 2021–2120. Cancer cases and deaths averted (health outcomes) are discounted at 0%. Only cervical cancer caused by HPV 16, 18, 31, 33, 45, 52 and 58, which could be averted by the 9-valent HPV vaccine, were considered. The lines represent the median projections of the 10 model-country settings: the HSA model in black, HPV-ADVISE models in red, and the Harvards models in blue. The grey area corresponds to the additional cases or deaths averted in the vaccinated cohorts after the 100 years of routine vaccination.

2.2 Cervical cancer cases and deaths: discounting health outcomes at 3%

A discount rate of more than 0% reduces the value of future health and assumes that present health is valued more than future health. This leads to benefits and costs being regarded as less important the further they arise in the future. Unlike curative therapies, vaccination programmes are usually sensitive to the choice of discount rates because most benefits accrue further into the future. The direct benefit of HPV vaccination is the prevention of HPV-related cancers (e.g., cervical cancer) in the vaccinated cohort, which is often realised several decades after immunisation. WHO-CHOICE and the WHO Guide on Standardization of Economic Evaluations of Immunization Programmes recommend using 0% and 3% per year on health outcomes and costs.

Figures 3 and 4 present the cervical cancers cases and deaths that could have been averted by routine one-dose HPV vaccination in 188 countries over the years 2021–2120. Cancer cases or deaths averted (health outcomes) are discounted at 3%. Only cervical cancer caused by HPV 16, 18, 31, 33, 45, 52 and 58, which could be averted by the 9-valent HPV vaccine, were considered. Figure 5 shows the cumulative cervical cancers cases that could have been averted by routine one-dose HPV vaccination in 188 countries over the years 2021–2120 when health outcomes are discounted at 3%.

Figures 6 and 7 present the cervical cancer cases and deaths averted by routine one-dose HPV vaccination programmes as a proportion of the cancers averted by a two-dose HPV vaccination programmes with a perfect vaccine (i.e., 100% vaccine efficacy) conferring lifelong protection. The median percentage (intervals: 10–90th percentile) of cancers not averted by a one-dose schedule compared to a two-dose program of the 10 model-country settings: the HSA model in black, HPV-ADVISE model-country pairs in red, and the Harvard model-country pairs in blue.

When we discount health benefits, the model predicts that routine vaccination would prevent fewer cancers and deaths, and more girls need to be vaccinated to avert one cervical cancer case (Figure 4 in main paper).

When we investigated the impact of a one-dose vaccination schedule with a 2-valent vaccine, the model predicts many cancers can still be averted by routine vaccination with one dose of the 2-valent vaccine (Figure 8). In 188 countries over the years 2021–2120, the models projected that routine annual vaccination of 10-year-old girls (plus a one-year catch-up campaign of girls aged up to age 14 years) with one dose of the 2-valent HPV vaccine at 80% coverage would avert 78.0 million (80%UI 54.2–93.7) and 100.4 million (80%UI 71.4–123.2) cervical cancer cases should one dose of the vaccine confer 20 and 30 years of protection, respectively (Figure 8). Under a scenario of one dose of the 2-valent vaccine providing lifelong protection at 80% initial VE, the models predicted that 104.8 million (80%UI 98.4–117.2) cervical cancer cases would be prevented (Figure 8). Figure 9 presents the cervical cancers cases that could have been averted by routine one-dose HPV vaccination in 188 countries over the years 2021–2120 when vaccination coverage is lowered to 40%. Figure 10 presents the cervical cancer cases averted by routine one-dose HPV vaccination programmes as a proportion of the cancers averted by a two-dose HPV vaccination programmes with a perfect vaccine (i.e., 100% vaccine efficacy) conferring lifelong protection when vaccination coverage is at 40%.

The start year of vaccination does not affect the comparison of one-dose against two-dose schedules. Figure 11 shows that there are very small changes in the proportion of cancers averted by a routine one-dose schedule

compared to a two-dose programme if routine vaccination is delayed by several years (e.g., 10 years to 2031 instead of 2021 for the UK HSA model). Figure 12 shows that the number of cervical cancers averted by routine one-dose schedules would not be changed substantially if routine vaccination is delayed by 10 years. The slight decrease in the number of cancers averted if routine vaccination is delayed, is simply an artefact of the fixed time horizon (2021–2120). However, fewer cancers are likely to be averted if routine vaccination is delayed because of missed cohorts.

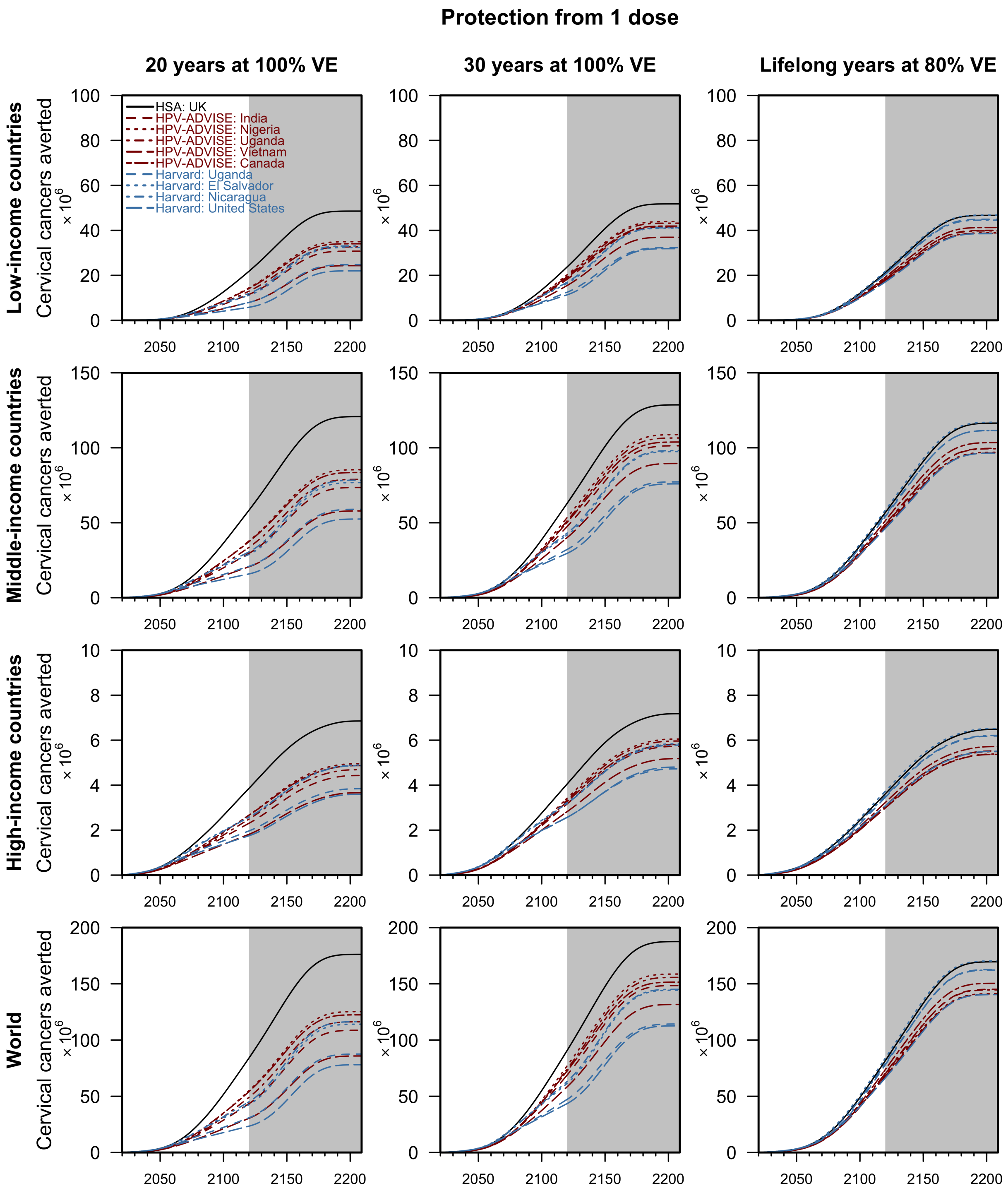

Figure 1: Cumulative cervical cancers averted by routine one-dose HPV vaccination by income groups, no discounting.

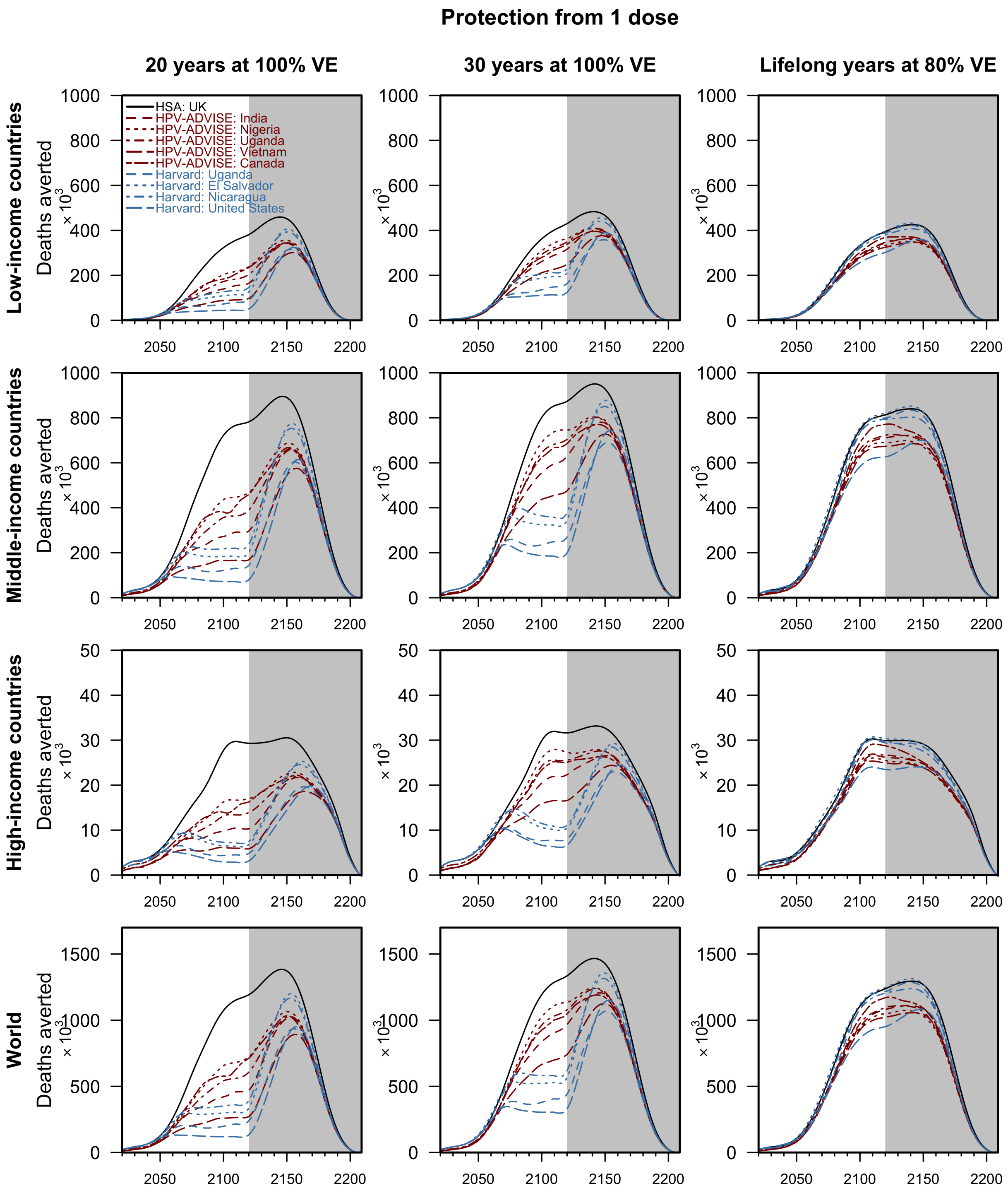

Figure 2: Cervical cancer deaths averted by routine one-dose HPV vaccination by income groups, no discounting.

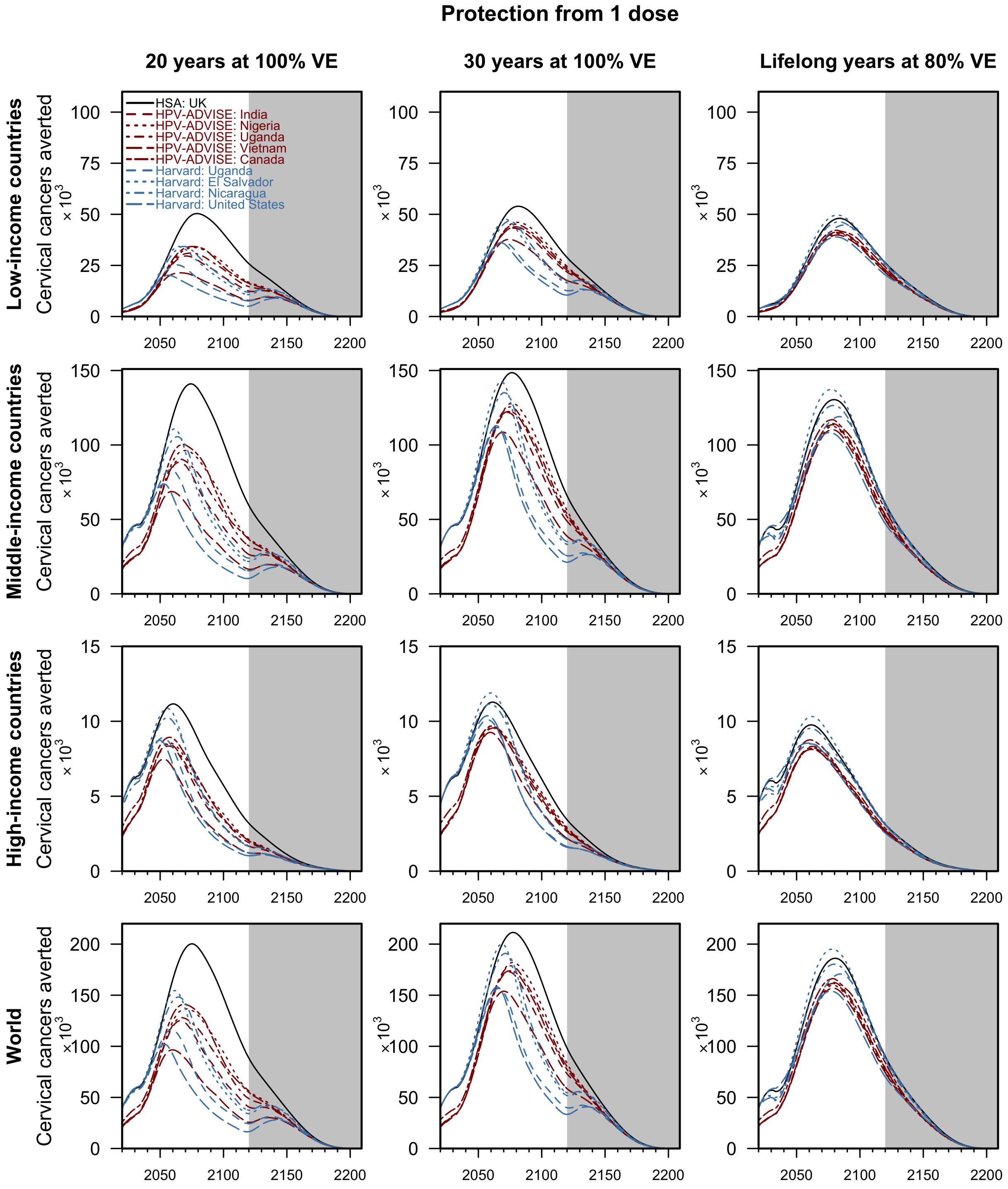

Figure 3: Cervical cancers averted by routine one-dose HPV vaccination by income groups, discounted.

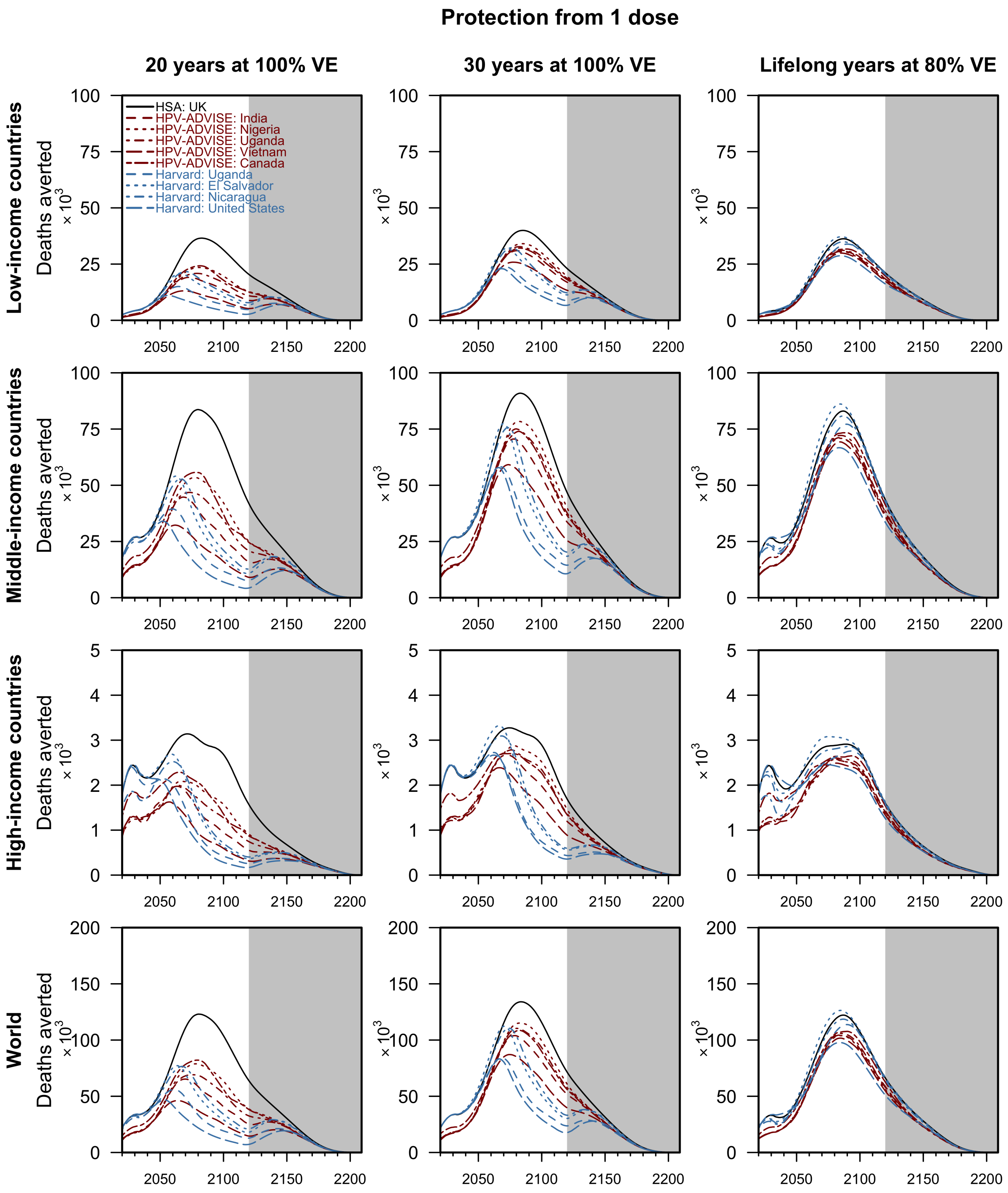

Figure 4: Cervical cancer deaths averted by routine one-dose HPV vaccination by income groups, discounted.

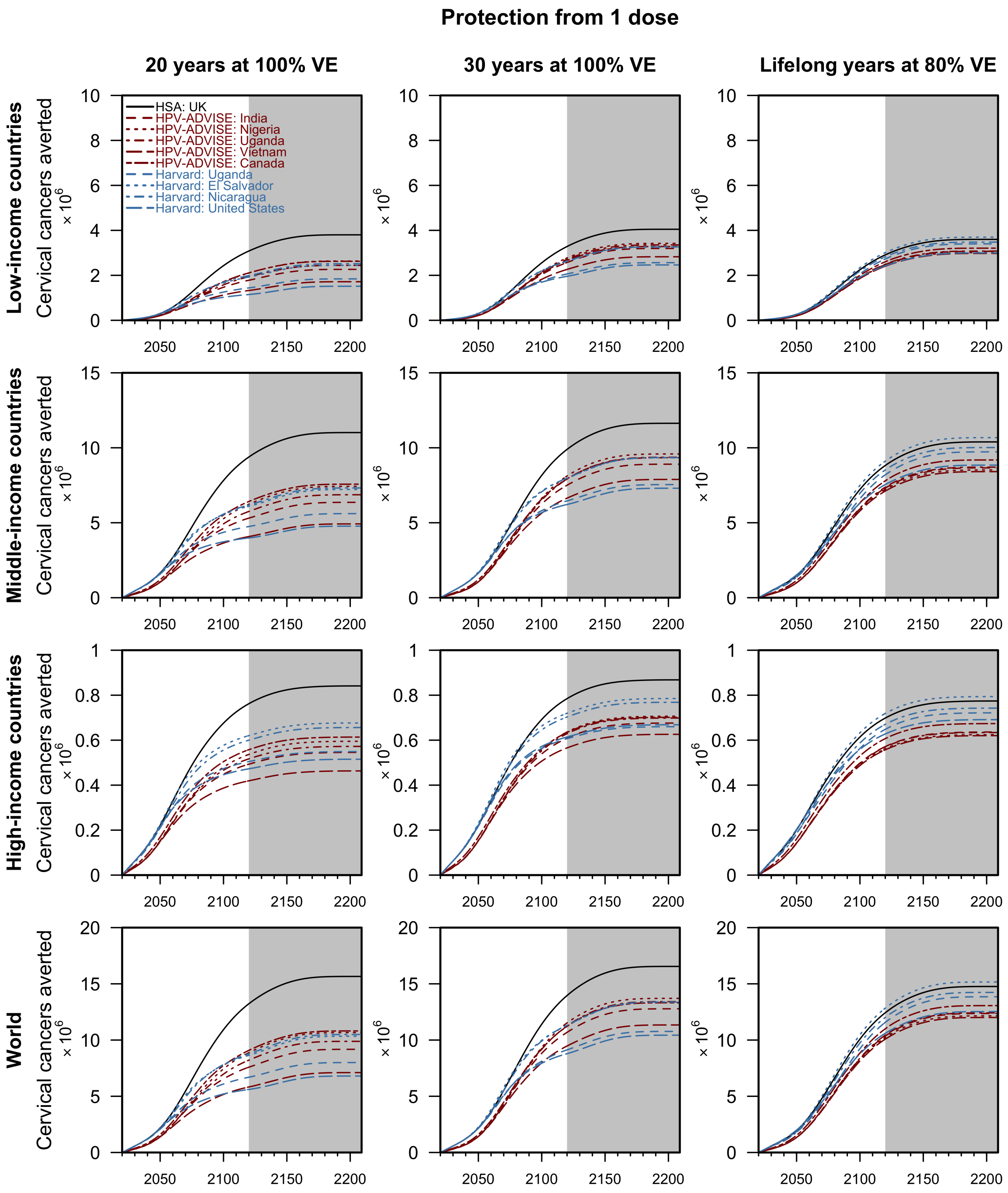

Figure 5: Cumulative cervical cancers averted by routine one-dose HPV vaccination by income groups, discounted.

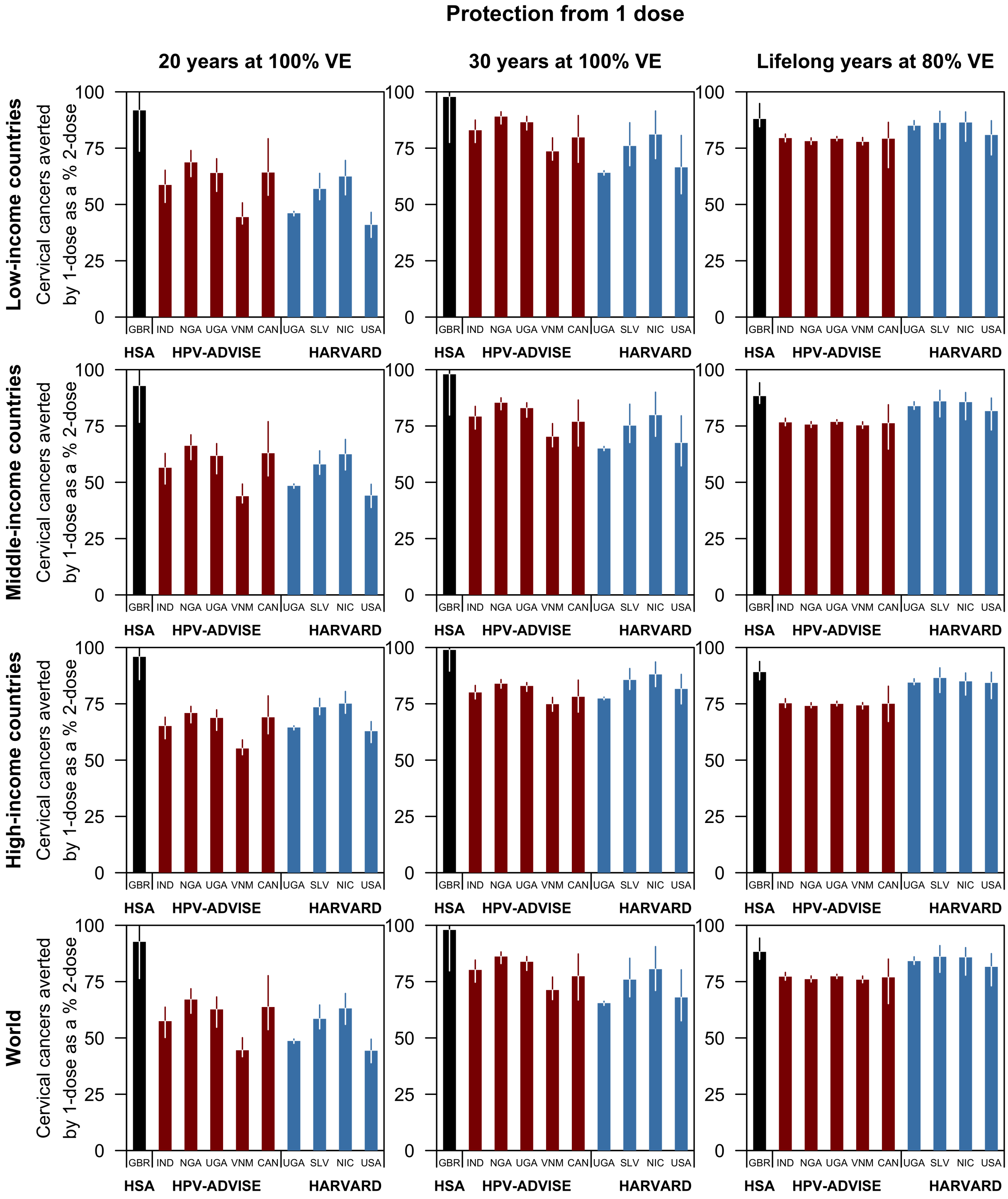

Figure 6: Proportion of cervical cancers averted by 1-dose compared to a perfect vaccine, discounted.

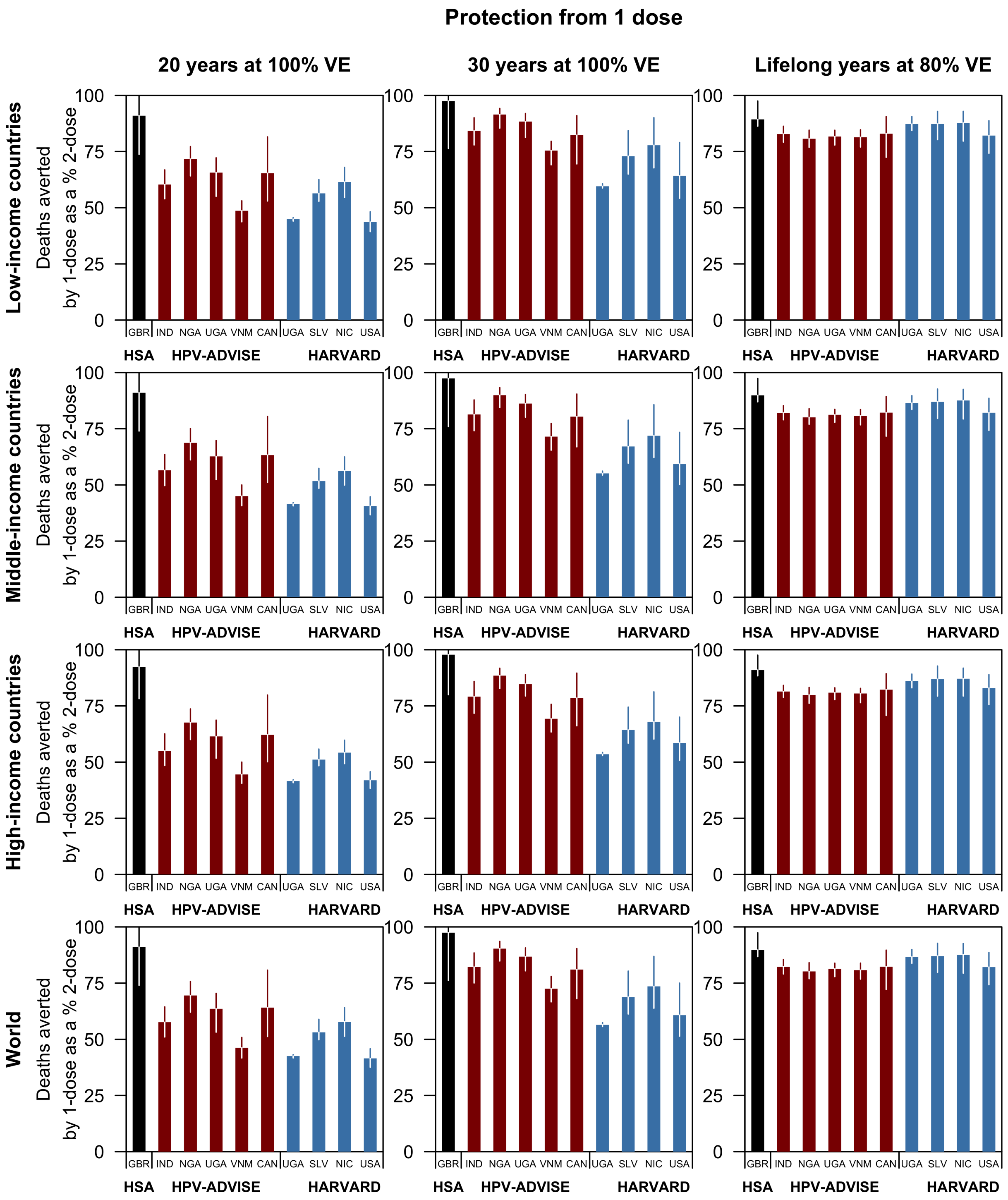

Figure 7: Proportion of cervical cancer deaths averted by 1-dose compared to a perfect vaccine.

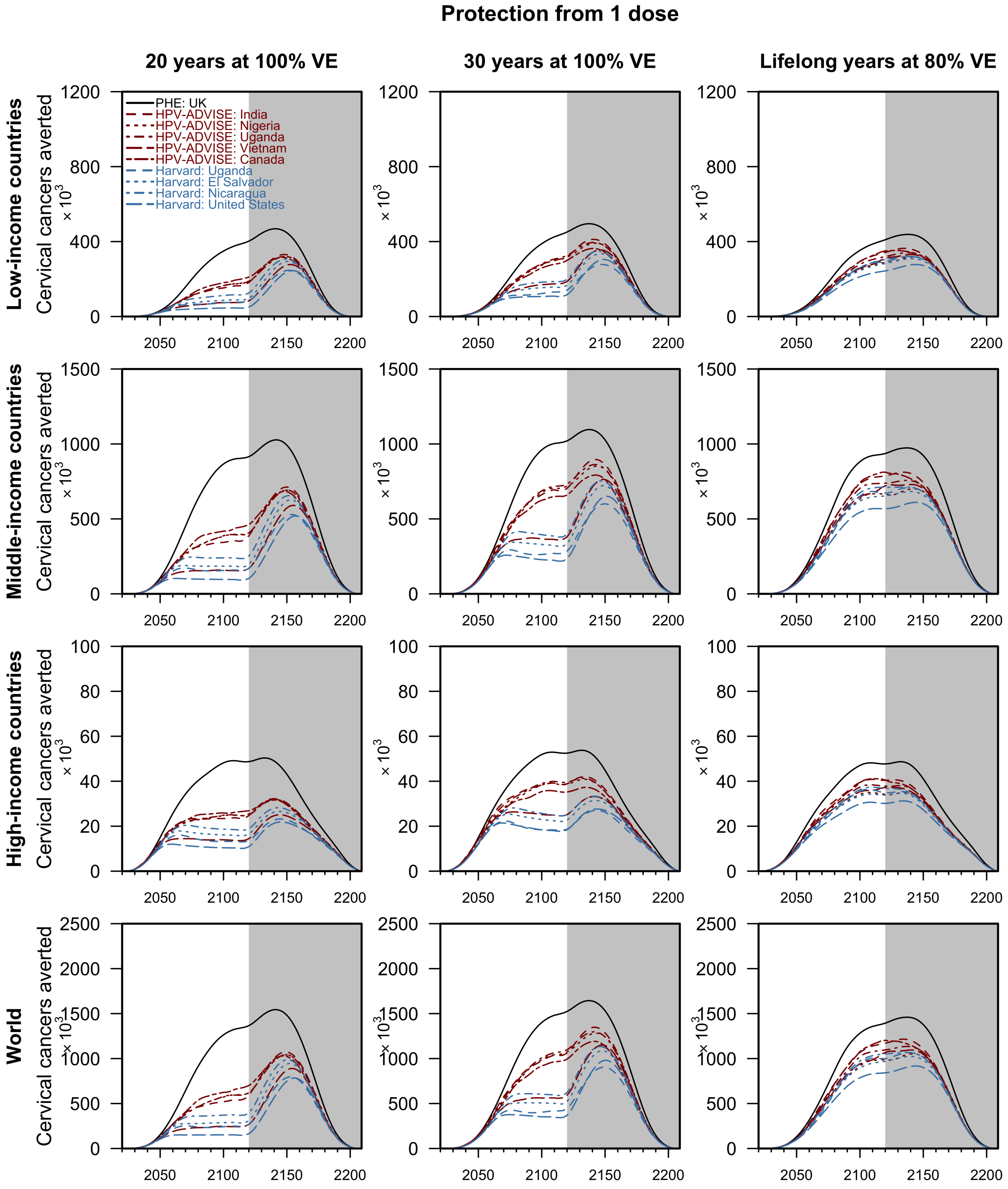

Figure 8: Cervical cancers averted by routine one-dose HPV vaccination by income groups with a 2-valent vaccine.

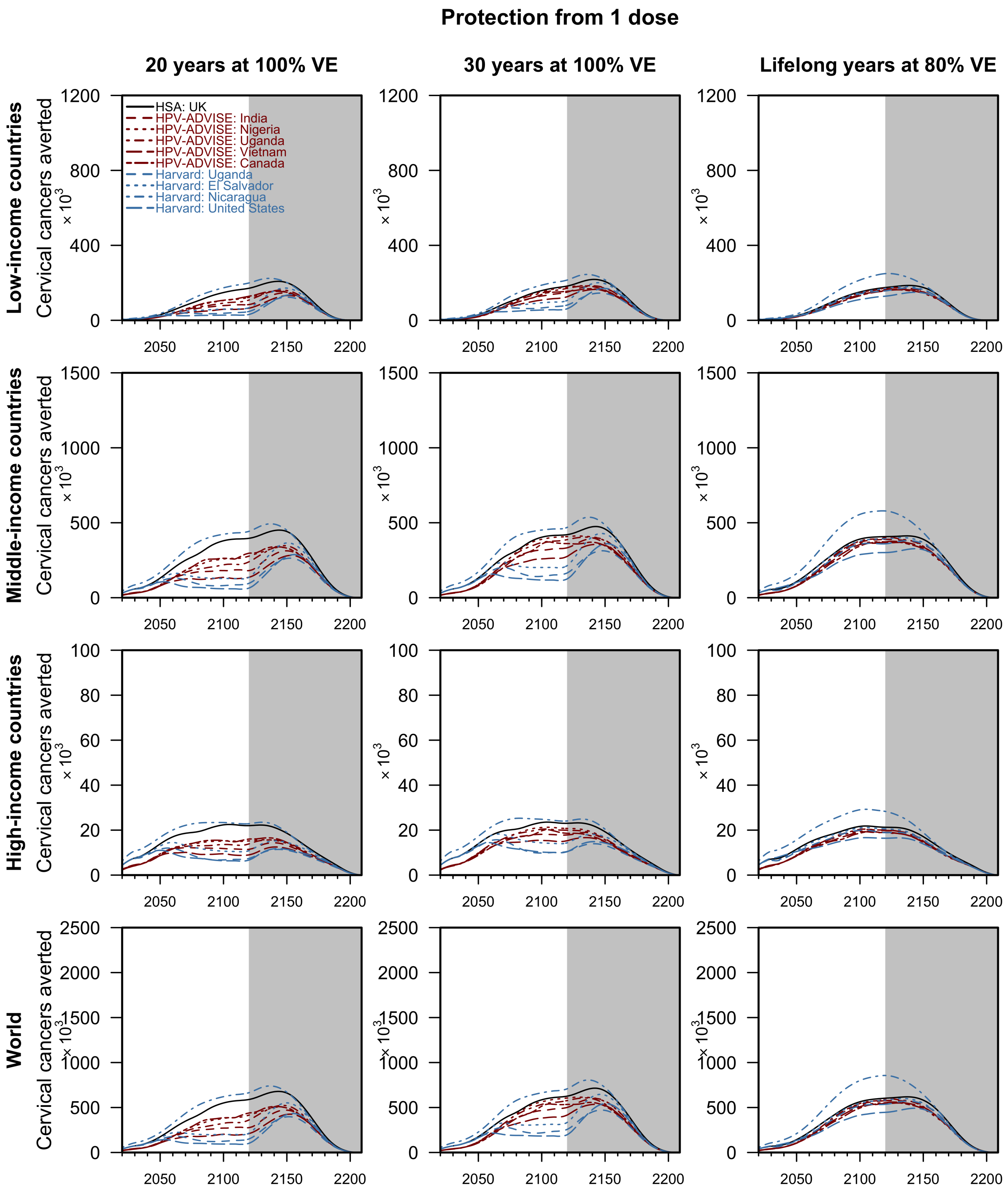

Figure 9: Cervical cancers averted by routine one-dose HPV vaccination by income groups at lower coverage.

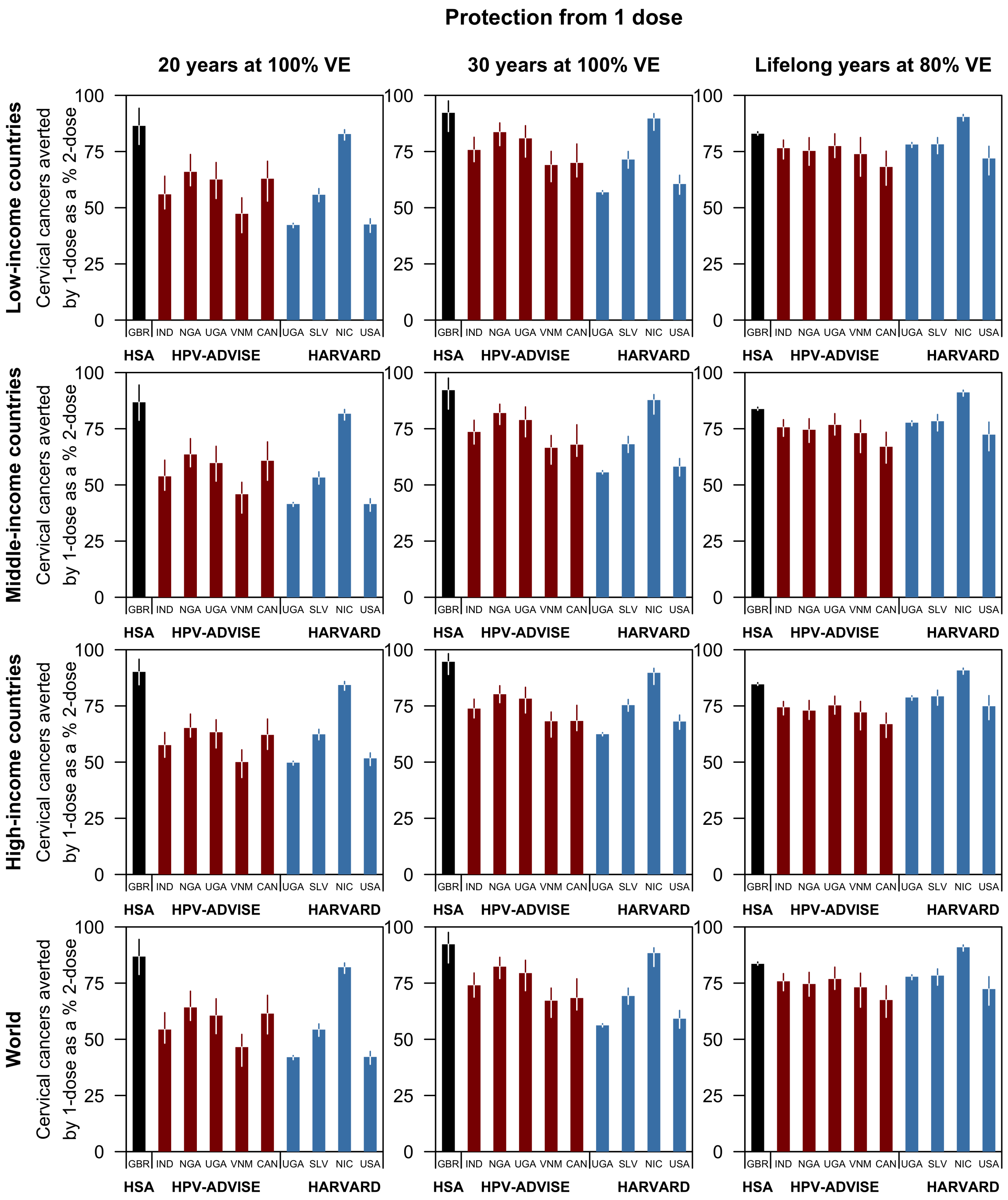

Figure 10: Proportion of cervical cancer deaths averted by 1-dose compared to a perfect vaccine at lower coverage.

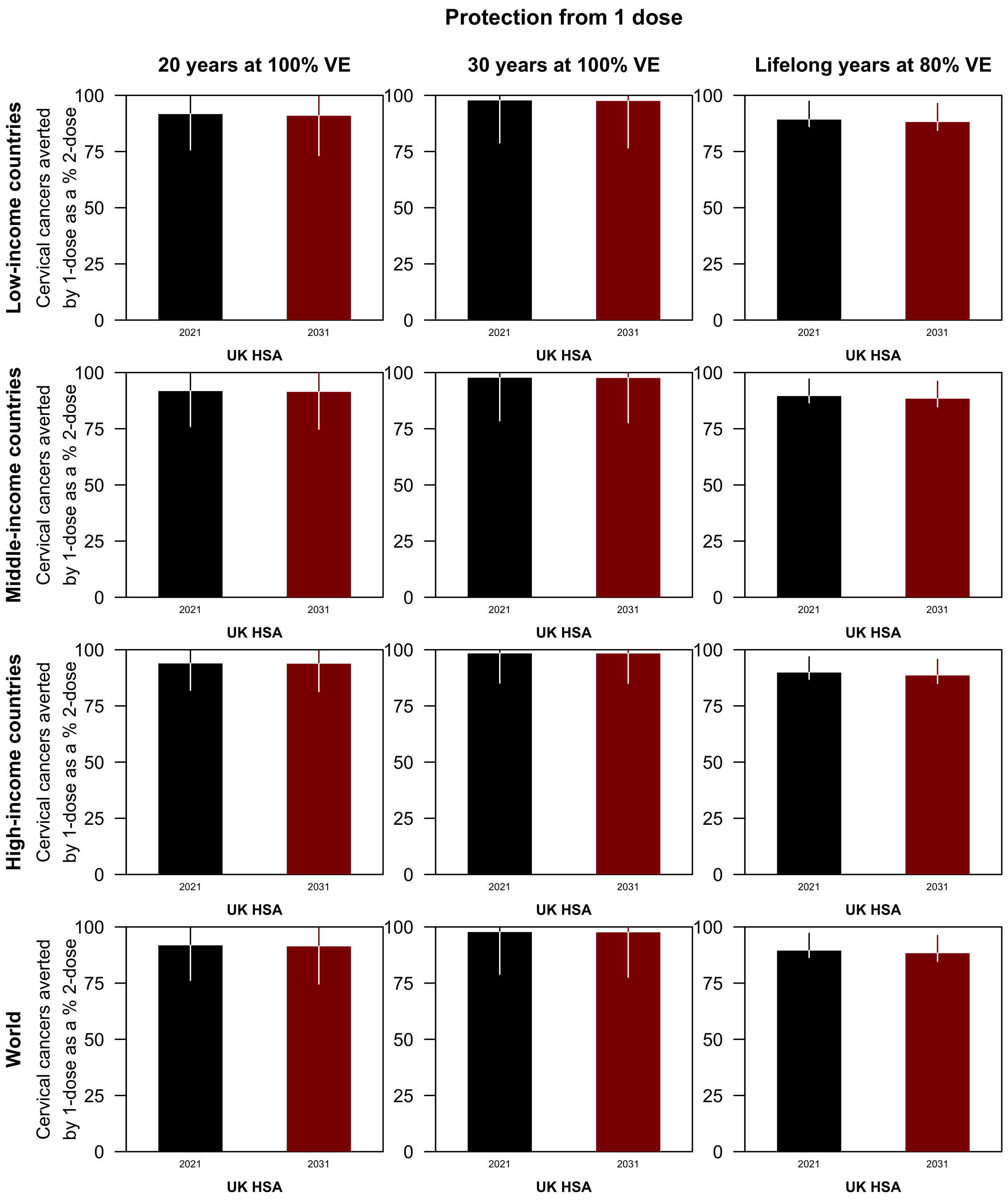

Figure 11: Proportion of cervical cancer averted by 1-dose compared to a perfect vaccine when vaccination is delayed.

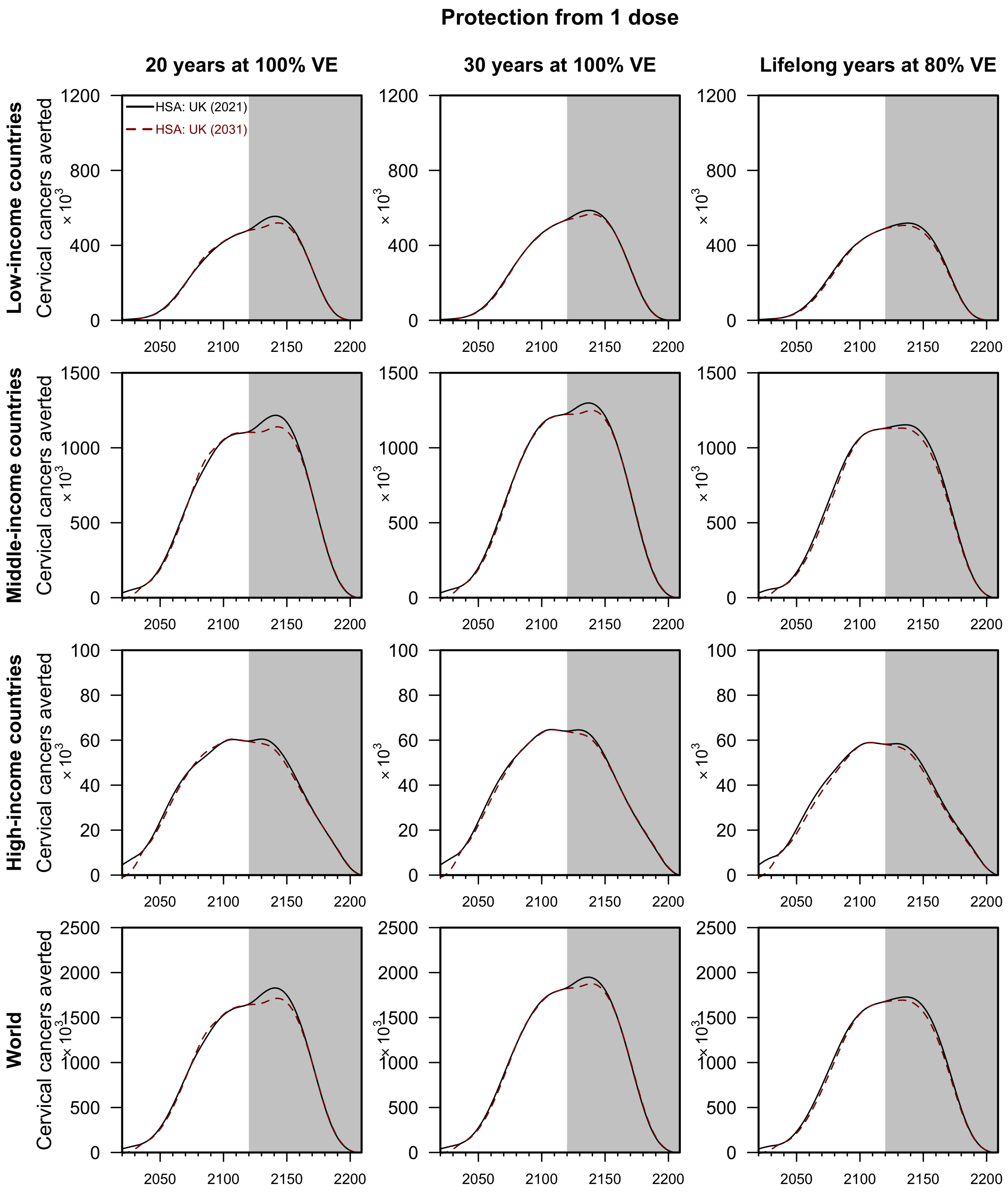

Figure 12: Cervical cancers averted by routine one-dose HPV vaccination by income groups at when vaccination is delayed.

2.3 Threshold costs

The threshold cost is the maximum that could be paid for the first dose (compared to no vaccination) and second dose (compared to one dose only) for the incremental cost-effectiveness ratio to remain below the cost-effectiveness threshold. Two cost-effectiveness thresholds are presented (Jit, 2020; Ochalek et al., 2020): country gross domestic product (GDP) per capita (in 2017 USD) costs in panels A–D and a lower threshold as suggested by Jit (2020). The lower cost-effectiveness threshold considered is 30–40% and 60–65% of GDP per capita in low-income and middle- to high-income countries, respectively. Both health outcomes and costs are discounted at 0% and 3%. We used the GDP per capita estimates by the World Bank.

Figure 13 presents the threshold cost for the first dose (compared to no vaccination) and second dose (compared to one dose only) under two cost-effectiveness thresholds: country gross domestic product (GDP) per capita (in 2017 USD) costs in panels A–D and a lower threshold as suggested by Jit (2020). The lower cost-effectiveness threshold presented in panels E–H is 30–40% and 60–65% of GDP per capita in low-income and middle- to high-income countries, respectively. Both cost and health outcomes are discounted at 3%. In Figure 14, both cost and health outcomes are not discounted.

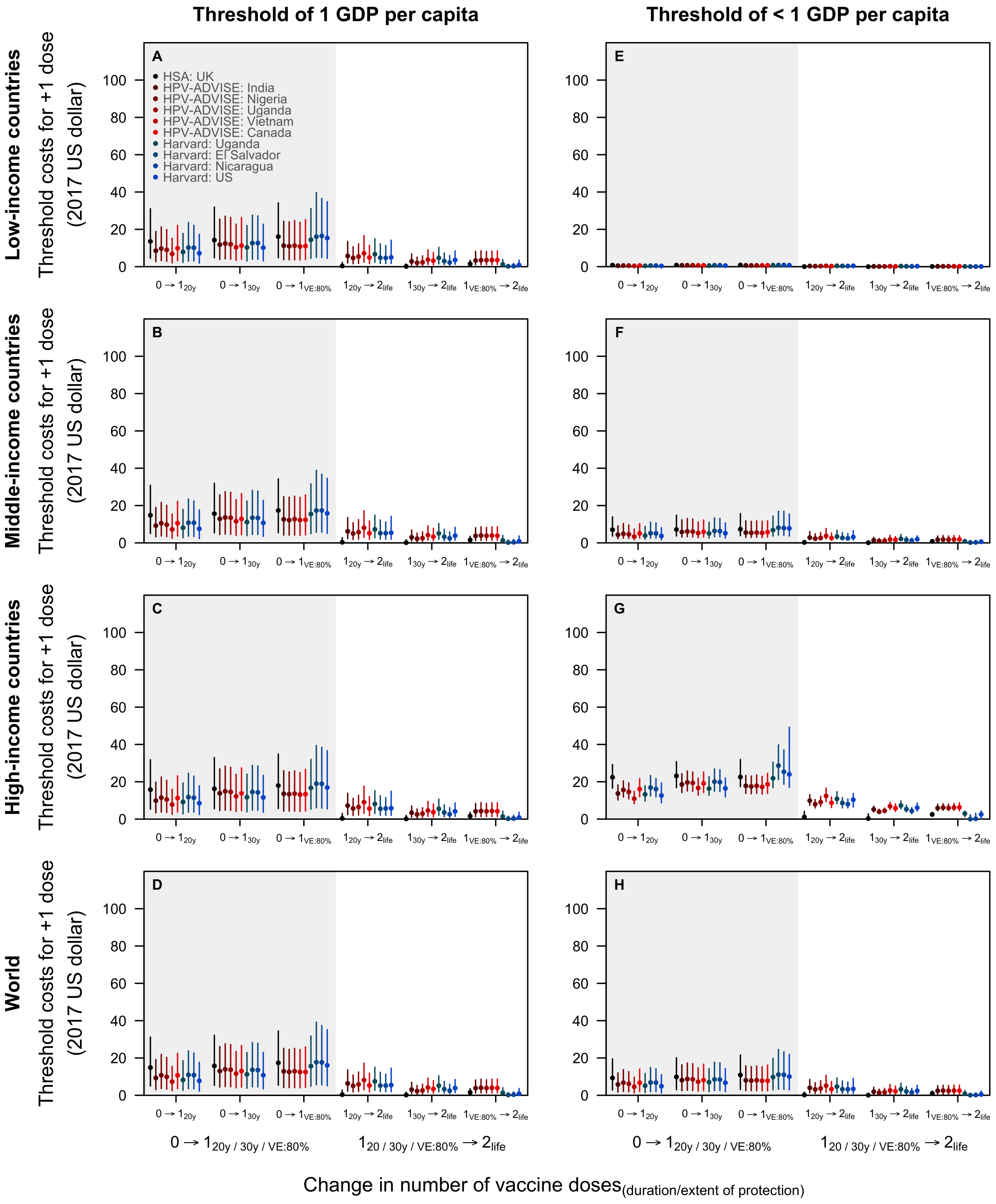

Figure 13: Threshold cost to pay for the first and second dose of vaccine, discounting on health outcomes and costs.

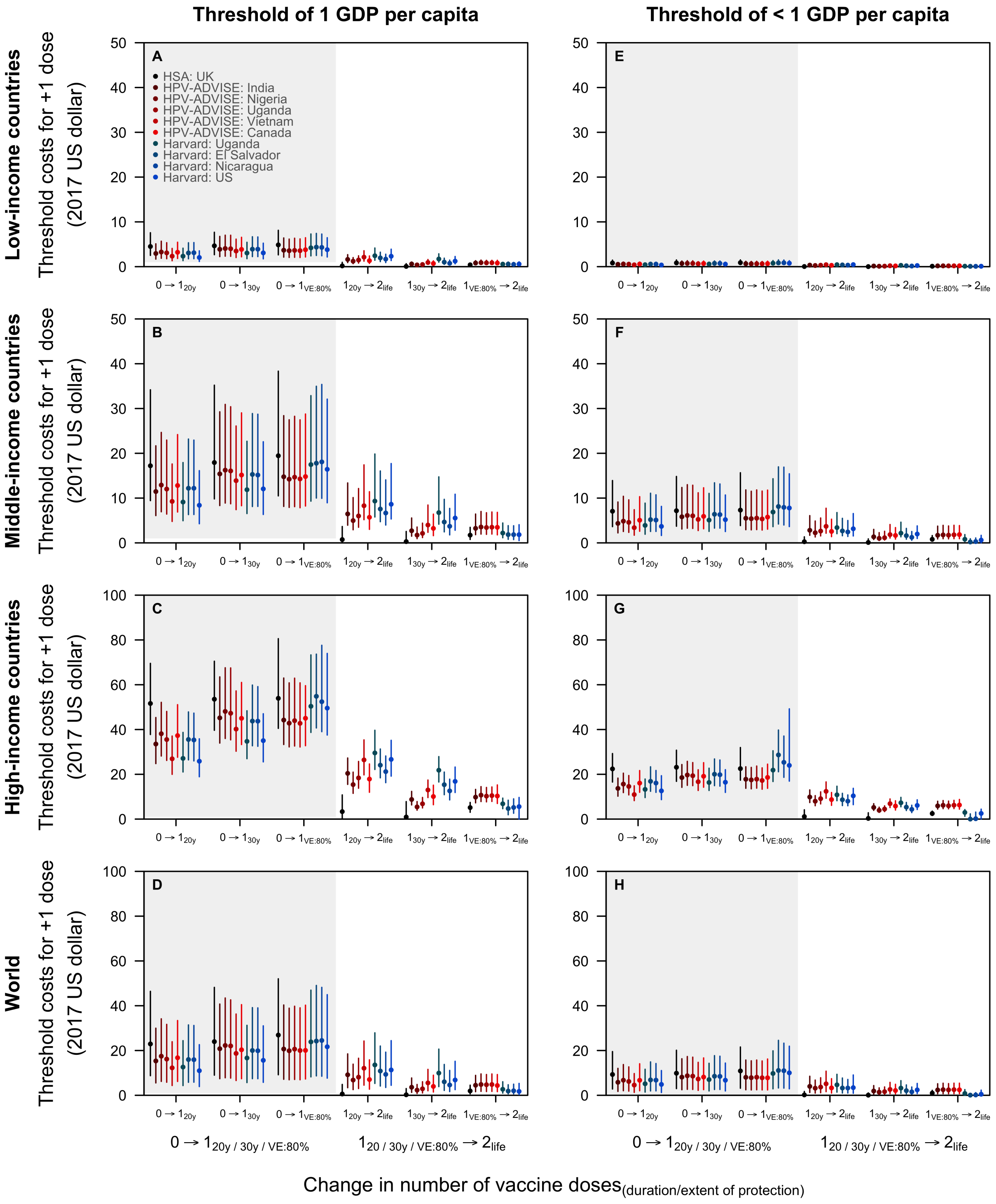

Figure 14: Threshold cost to pay for the first and second dose of vaccine, no discounting.

2.4 Number needed to vaccinate

The time horizon of the analysis is from 2021 to 2120. However, we accrue all health benefits of vaccination up to the end of the routine vaccination programme (i.e. the year 2120, in the figure below) or age 100 of all vaccinated cohorts. Over the years 2021 to 2120, Figure 15 shows the number of girls needed to be vaccinated with the first and second dose to avert one additional cervical cancer case by income group when health outcomes are discounted at 3% (panels A–D) and 0% (panels E–H). The lines represent the median projections of the nine models: the HSA model in black, HPV-ADVISE models in red, and the Harvards models in blue. Health outcomes are discounted at 3% (panels A–D) and 0% (panels E–H). In the main paper, we present the results when we accrue health benefits of vaccination up to age 100 of all vaccinated cohorts.

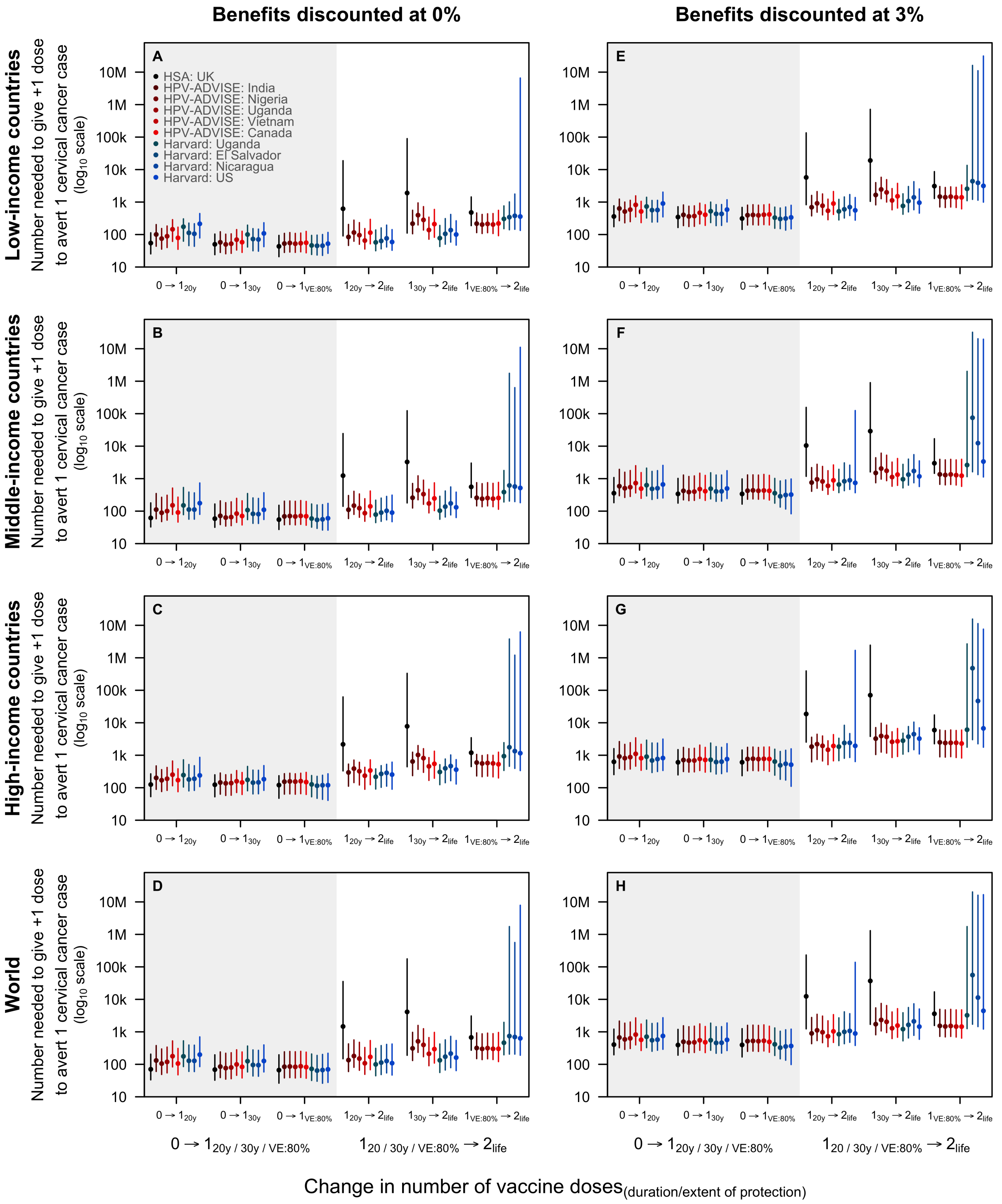

Figure 15: Number of girls needed to be vaccinated to avert one additional case over the years 2021–2120.

2.5 Internal validation

We performed internal validation to ensure the projection procedure did not distort the model results from the (source) dynamic models, which serve as inputs to the global model. The global estimates of vaccine impact were compared to those projected two dynamic models fitted to Uganda data: HPV-ADVISE and Harvard.

The Harvard model used the median age- and year-specific rates of cervical cancer per 100 000 projected by the Uganda-fitted Harvard model scaled to the Uganda population projections. The HPV-ADVISE model used the mean age- and year-specific cervical cancer rates per 100 000 projected by the Uganda-fitted HPV-ADVISE model scaled to the Uganda population projections. Total cases expected across the age groups were accumulated and summed from 2020-2120 for the natural history and the four vaccination scenarios. We calculated the cervical cancers averted compared with the natural history scenario, as well as the proportion of cases averted by the 1-dose vaccination scenarios compared to the 2-dose scenario.

Table 7: Internal validation

% cancers averted by 1-dose vs perfect vaccine	**Harvard** Uganda	**Global**	**HPV-ADVISE** Uganda	**Global**
**1-dose vaccine scenarios**				
9-valent at coverage 80%				
20y protection	36	47	59	66
30y protection	56	61	88	88
Lifelong at 80% VE	85	87	81	81
9-valent at coverage 40%				
20y protection	31	42	58	61
30y protection	50	56	86	79
Lifelong at 80% VE	76	78	80	77
2-valent at coverage 80%				
20y protection	35	46	59	64
30y protection	55	60	88	88
Lifelong at 80% VE	84	85	81	82

The internal validation (presented in Table 7) found that the global impact projections were close to those generated by two dynamic models for Uganda.
